# Aerosol and intranasal delivery of monoclonal antibodies to prevent transmission in pig influenza infection models

**DOI:** 10.1038/s41541-025-01277-9

**Published:** 2025-11-05

**Authors:** Catherine F. Hatton, Emily Briggs, Eleni Polychronakis, Ashutosh Vats, Bhawna Sharma, Sanis Wongborphid, Tiphaine Cayol, Ehsan Sedaghat-Rostami, Shathviga Manoharan, Alice Guan, Elliot J. Moorhouse, Ronan MacLoughlin, Pramila Rijal, Alain R. Townsend, Francisco J. Salguero, Basudev Pauydal, Elma Tchilian

**Affiliations:** 1 The Pirbright Institute, Pirbright, UK; 2 The University of Surrey, Guildford, UK; 3 The University of Manchester, Manchester, United Kingdom; 4 Aerogen Ltd, Galway, Ireland; 5 Chinese Academy of Medical Science Oxford Institute, Nuffield Department of Medicine, University of Oxford, Oxford, UK; 6 MRC Human Immunology Unit, MRC Weatherall Institute of Molecular Medicine, John Radcliffe Hospital, University of Oxford, Oxford, UK; 7 United Kingdom Health Security Agency, UKHSA-Porton Down, Salisbury, UK

**Keywords:** Influenza virus, Influenza virus, Antibodies

## Abstract

There is an urgent need for robust animal models to assess novel therapies that prevent the transmission of respiratory pathogens. We developed two complementary pig influenza models, direct and contact challenge, to evaluate the ability of monoclonal antibodies to block transmission. Using the strongly neutralizing 2–12 C mAb targeting H1N1pdm09 haemagglutinin, we established a benchmark for comparing mAb delivery routes and platforms. Intravenous administration of 2–12 C consistently showed the highest efficacy in the direct challenge model. The contact influenza challenge model, which best mimics natural exposure, was further optimized by evaluating key parameters, including timing of co-housing, infectious dose, and delivery routes. Aerosol and intravenous delivery of 2–12 C were equally potent, preventing infection in contact animals, while intranasal delivery prevented infection in some but not all animals. The pig direct and contact influenza challenge models provide powerful platforms for the evaluation therapeutic strategies to prevent influenza disease and transmission in humans.

## Introduction

Respiratory infections, particularly influenza, remain a significant global threat to both animals and humans, contributing substantially to morbidity and mortality. Current immunization strategies, although effective in preventing severe disease, reducing hospitalizations and deaths, do not completely prevent transmission. Therefore, novel therapeutic approaches are needed to limit transmission and protect individuals at risk of respiratory disease, including those who are unvaccinated, unable to mount an adequate immune response, or experiencing breakthrough infections despite full vaccination.

Neutralizing monoclonal antibodies (mAbs) represent a promising strategy for preventing and treating influenza. The influenza hemagglutinin (HA) protein facilitates viral entry by binding to sialic acid on host cells. Antibodies targeting HA have been shown to limit disease progression in animal models^1–3^. However, parenteral administration may result in suboptimal bioavailability in the respiratory tract (RT), making mucosal delivery a potentially more effective route for preventing infection and transmission of respiratory viruses. Inhaled mAb delivery offers direct targeting of the airways. The large lung surface area ( ~100 m^2^), thin airway epithelium, and rich vascularization promote rapid absorption and fast mAb availability. Furthermore, significantly lower doses are required to achieve therapeutic effects^4^.

Many studies demonstrate the efficacy of inhaled mAbs. The potent neutralizing mouse mAb 30D1, which targets the HA of pandemic H1N1 (H1N1pdm09), prevented transmission in guinea pigs and ferrets when administered intranasally as either IgG or IgA but not intramuscularly^5^. Similarly, the human IgG1 mAb CR911, targeting a conserved epitope on the HA stem domain, fully protected mice against low dose H5N1 infection following intranasal administration^6^. The C179 mAb conferred protection against lethal H5N1 and pH1N1 challenges^7^. Intranasal delivery of antibody combination CF-404 of three broadly neutralising mAbs prevented weight loss and death in mice challenged with either H1N1, H3N2, B/Victoria-lineage, or B/Yamagata-lineage influenza viruses^8^. The COVID-19 pandemic stimulated further research into inhaled mAbs for respiratory infections. For example, the mAb combination A8G6, targeting distinct SARS-CoV-2 epitopes, demonstrated good tolerability and delayed infection in humans when administered as a nasal spray and several other mAbs for respiratory pathogens have been tested in clinical trials^4,9,10^. These preclinical and clinical studies demonstrate the potential of inhaled mAbs as a powerful strategy for combating influenza and other respiratory pathogens.

However, all currently approved monoclonal antibodies (mAbs) are administered parenterally, while challenges remain for respiratory delivery^11^. Challenges associated with mucosal delivery include developing stable formulations that retain antibody efficacy during storage and inhalation, overcoming anatomical and biological barriers in the RT, and the lack of relevant animal models that fully replicate human lung anatomy and breathing patterns. For example, respiratory administration of an anti-respiratory syncytial virus (RSV) mAb, designed for systemic administration failed to prevent RSV infection in preterm infants^12^. Preclinical studies demonstrating the efficacy of intranasal mAb delivery have typically used large-volume bolus administrations in small animals, which may not accurately reflect human RT exposure. To address this, we used pigs, a large, natural influenza host with significant anatomical and physiological similarities to humans^13^, to analyze droplet distribution in the RT following intranasal delivery via a mucosal atomization device (MAD) or aerosol delivery using a vibrating mesh nebulizer (VMN)^14^. A scintigraphy study using Technetium-99m-labeled diethylenetriaminepentaacetic acid (99mTc-DTPA) showed that VMN administration resulted in a uniform droplet distribution throughout both the upper and lower RT. In contrast, MAD delivery (1 ml per nostril) deposited a significant portion of the dose in the lungs but showed more localized and uneven distribution^14^.

Using this pig model, we have shown that intravenous administration of the strongly neutralizing human mAb 2–12 C^15^, which targets the HA head, significantly reduced viral load and lung pathology following pandemic H1N1pdm09 (pH1N1) challenge^16,17^. While viral shedding was significantly reduced, it was not eliminated, likely due to the high viral challenge dose administered directly to the RT. However, intravenous administration of 2–12 C at 15 mg/kg completely prevented infection in a contact challenge model, where treated pigs were co-housed with donor pigs previously infected with pH1N1^17^. Interestingly, when 2–12 C was administered by VMN, nasal viral shedding was not significantly reduced following direct influenza challenge, although lung pathology and viral load in the lungs were markedly decreased.

These findings demonstrate the need to optimize mAb delivery strategies to the RT in large animal models and humans to effectively prevent influenza infection and transmission. In this study, we established robust pig direct and contact influenza challenge models to precisely characterise the optimal mAb dose, administration route, viral challenge dose, and timing of co-housing. These models provide valuable platforms for evaluating mAb delivery systems and other transmission-blocking therapeutics in a physiologically relevant large animal model.

## Results

### Efficacy of different doses and routes of administration of 2-12 C in direct influenza challenge model

**Fig. 1:**
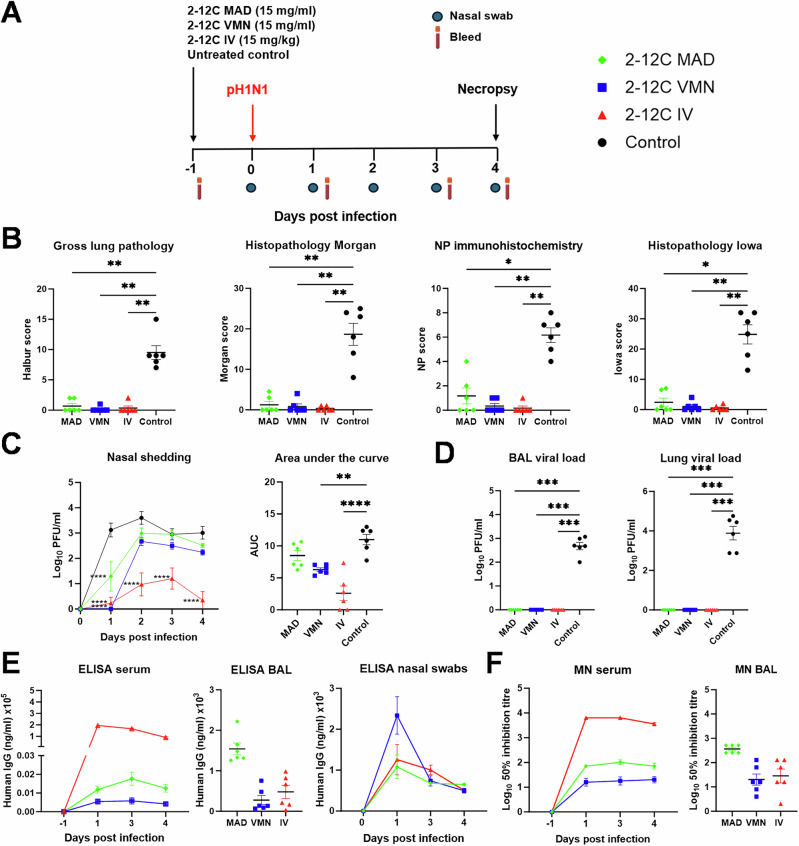
Experiment 1, direct challenge. **A** Schematic of experimental design. 2-12 C mAb was administered either intranasally by mucosal atomisation device (MAD), by aerosol using a vibrating mesh nebuliser (VMN) or intravenously (IV) to pigs. The control pigs were left untreated. After 24 h, all pigs were inoculated with pH1N1. Nasal swabs and serum were collected at the indicated time points. Necropsy was performed at 4 days post infection. **B** Lung pathology: gross lung pathology, histopathology Morgan, nucleoprotein (NP) immunohistochemistry and histopathology Iowa scores at day 4 post infection. **C** Viral titres in nasal swabs over time and associated area under the curve (AUC). **D** Viral titres in bronchoalveolar lavage (BAL) fluid and accessory lung lobe determined by plaque assay at 4 days post infection. **E** ELISA titres at specified time in serum, BAL fluid (day 4 post infection) and nasal swabs. **F** Neutralisation activity determined by microneutralisation (MN) assay of serum over time and BAL fluid at day 4 post infection. Each data point represents an individual pig (n = 6 per group) along with the mean ± SEM. Data sets were first assessed for normality and then subjected to either an ordinary one-way or two-way ANOVA test with a Dunnett’s multiple comparisons test or a Kruskal-Wallis test with a Dunn’s multiple comparisons test if they did not pass the normality test. Asterisks denote significance between indicated groups (*p < 0.05, ** p < 0.01, *** p < 0.001, **** p < 0.0001).

We have previously shown that intravenous (IV) delivery of 2-12 C effectively reduces viral load and lung pathology following intranasal pH1N1 challenge^16^. However, while viral shedding was reduced, it was not eliminated. We hypothesized that mucosal administration of 2-12 C at the site of viral entry might enhance efficacy. To compare IV and mucosal delivery, twenty-four pigs were randomized into four groups of six and 2-12 C administered either IV or intranasally using a mucosal atomization device (MAD), or by aerosol using a VMN (Fig. 1A). Control pigs were left untreated. MAD delivery generated 80–100 µM droplets which reach the lower respiratory tract when delivered in 1.5 ml per nostril^14^. VMN produced ~4 µm droplets capable of reaching the entire respiratory tract^14^. Twenty-four hours post-administration, pigs were infected intranasally with pH1N1 by MAD. Four days post-challenge, animals were culled, and blood, bronchoalveolar lavage (BAL), and lung tissues were collected to assess viral load and 2-12 C titres. This time point was chosen to allow monitoring of viral shedding in daily nasal swabs while ensuring significant viral load and pathology in the lungs.

Lung pathology (gross and histological) was significantly reduced in 2-12C-treated pigs compared to controls (Fig. 1B). Areas of broncho-interstitial pneumonia were present mainly in the cranial and middle lung lobes of animals from the control group, but no lesions were found in the 2-12 C treated groups. Necrotising bronchiolitis and bronchial and alveolar exudation, together with thickening of the alveolar septa, was detected in the animals from control group. Influenza A nucleoprotein (NP) was detected by immunohistochemistry (IHC) in the bronchial and bronchiolar epithelial cells, alveolar cells, and exudate within the bronchiolar and alveolar luminae in the control groups. One animal each from the IV and VMN 2-12 C treated groups showed NP labelling.

IV administration led to the greatest reduction in viral shedding, while MAD and VMN significantly reduced shedding only on day 1. However, VMN and IV significantly reduced overall shedding (AUC) compared to controls (Fig. 1C). No virus was detected in BAL or lung tissue of any of the 2-12 C treated animals (Fig. 1D).

Serum 2-12 C concentrations at -1, 1, 3, and 4 DPI, measured by ELISA, peaked at 1 DPI in the IV group and was ~90 μg/ml at 4 DPI, whereas MAD and VMN groups exhibited ~two logs lower levels (1.25 μg/ml for MAD and 0.42 μg/ml VMN) at 4 DPI (Fig. 1E). 2-12 C titers in BAL were highest in the MAD group at an average of 1.54 μg/ml at 4 DPI, although not statistically different compared to VMN and IV groups. 2-12 C was also detected in nasal swabs in all treatment groups. Neutralizing activity in serum and BAL mirrored 2-12 C ELISA titres (Fig. 1E, F; Table 1).

**Table 1:** Experimental design, lung pathology, viral load and 2-12 C titres in serum, BAL fluid and nasal swabs

Experiment number	Challenge	Rec mAb 2-12 C route and dose/or dose of virus	Gross lung pathology vs control	AUC nasal shedding vs control	BAL viral load vs control	Lung viral load vs control	ELISA titre serum (μg/ml)^a^	ELISA titre BAL (μg/ml)^a^	ELISA titre nasal swabs (μg/ml)^b^
1	Direct	MAD 15mg/ml (3 ml) VMN 15 mg/ml (4 ml) IV 15 mg/kg Control	Sig ** Sig ** Sig **	NS Sig ** Sig ****	Sig *** Sig *** Sig ***	Sig *** Sig *** Sig ***	1.25 ± 0.21 0.42 ± 0.08 89.7 ± 7.6	1.54 ± 0.15 0.28 ± 0.11 0.48 ± 0.16	1.37 ± 0.03 2.83 ± 0.42 1.02 ± 0.42
2	Direct	VMN 40 mg/ml (4 ml) IV 25 mg/kg Control	Sig ** Sig **	Sig * Sig *	NS Sig *	NS Sig *	0.24 ± 0.04 214.7 ± 22.7	1.84 ± 1.5 0.3 ± 0.07	1.8 ± 0.8 0.5 ± 0.2
3	Contact (6 days)	IV 14 mg/kg Control	Sig *	Sig **	ND	ND	26.5 ± 4.5	0.29 ± 0.15	0.19 ± 0.01
4	Contact (3 days)	IV 25 mg/kg Control	Sig ***	Sig ***	Sig ****	Sig ****	41.5 ± 5.1	0.38 ± 0.3	0.32 ± 0.02
5	5a. Contact (3 days) 5b. Contact 2 days	MAD 25 mg/ml (3 ml) VMN 25 mg/ml (3 ml) IV 15 mg/kg 3 DPC control 2 DPC control	Sig * Sig * Sig * NS	Sig * Sig ** Sig ** NS	Sig ** Sig ** Sig ** NS	Sig **** Sig **** Sig **** NS	0.22 ± 0.06 0.25 ± 0.04 32.9 ± 2.5 NA	0.11 ± 0.03 0.33 ± 0.06 0.06 ± 0.01 NA	0.18 ± 0.02 1.85 ± 0.71 0.43 ± 0.13 NA
6	Contact (2 days)	Control (10^6^ PFU) Control (10^4^ PFU) Control (10^3^ PFU) Control (2-12 C IV 10 mg/kg +10^6^ PFU)	NA	NA	NA	NA	NA	NA	NA
7	Direct	Control (1 ml 10^4^ PFU) Control (0.5 ml 10^4^ PFU) Control (0.25 ml 10^4^ PFU)	NA	NA	NA	NA	NA	NA	NA

Significance is determined by comparison to the untreated control *NS* nonsignificant, *ND* not determined, *NA* not applicable, *BAL* bronchoalveolar lavage, *PFU* plaque-forming units, *SEM* standard error of the mean. ^a^Average value at day of cull or ^b^ at 1 DPI/DPC ± SEM. Data sets were first assessed for normality and then subjected to an unpaired *t* test. Asterisks denote significance between indicated groups (*p < 0.05, **p < 0.01, ***p < 0.001, ****p < 0.0001).

**Fig. 2:**
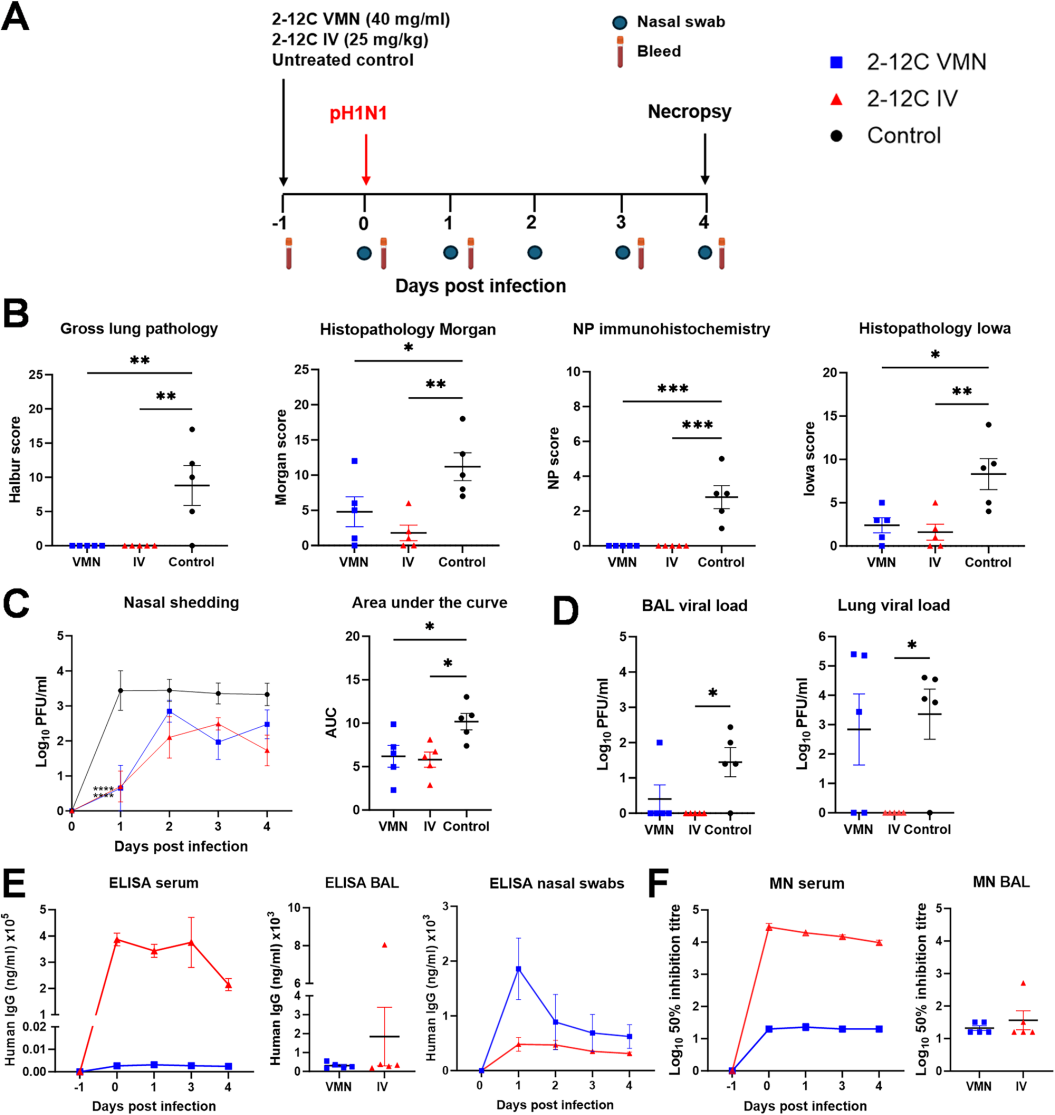
Experiment 2, direct challenge. **A** Schematic of experimental design. 2-12 C mAb was administered either intranasally by aerosol using a vibrating mesh nebuliser (VMN) or intravenously (IV) to pigs. The control pigs were left untreated. After 24 h, all pigs were inoculated with pH1N1. Nasal swabs and serum were collected at the indicated time points. Necropsy was performed at 4 days post infection. **B** Lung pathology: gross lung pathology, histopathology Morgan, nucleoprotein (NP) immunohistochemistry and histopathology Iowa scores at day 4 post infection. **C** Viral titres in nasal swabs over time and associated area under the curve (AUC). **D** Viral titres in BALand accessory lung lobe determined by plaque assay at 4 days post infection. **E** ELISA titres at specified time in serum, BAL fluid (day 4 post infection) and nasal swabs. **F** Neutralisation activity determined by microneutralisation (MN) assay of serum over time and BAL fluid at day 4 post infection. Each data point represents an individual pig along (n = 5 per group) with the mean ± SEM. Data sets were first assessed for normality and then subjected to either an ordinary one-way or two-way ANOVA test with a Dunnett’s multiple comparisons test or a Kruskal-Wallis test with a Dunn’s multiple comparisons test if they did not pass the normality test. Asterisks denote significance between indicated groups (*p < 0.05, **p < 0.01, *** p < 0.001).

Because viral shedding was not prevented following IV and VMN delivery, we increased the dose to 25 mg/kg IV and 40 mg/mL by VMN in 4 ml volume (Fig. 2A). As before, lung pathology was significantly reduced in the 2-12C-treated animals (Fig. 2B). Viral shedding in the IV and VMN groups was comparable and significantly lower than in the control group, although again shedding was not eliminated. Interestingly, the higher IV dose of 25 mg/kg did not further reduce viral shedding compared to 15 mg/kg (Figs. 1C and 2C). This time, virus was detected in the BAL and lung samples of the VMN group. Higher 2-12 C ELISA and neutralization titres were observed in serum of 25 mg/kg IV treated pigs compared to those treated with 15 mg/kg (Fig. 1E, F, Table 1). 2-12 C titers were also detected in nasal swabs in both VMN and IV groups. The BAL titres were comparable between IV and VMN groups (Fig. 2E, F, Table 1).

Taken together these results indicate that prophylactic administration of 2-12 C at 15 mg/kg or 25 mg/kg significantly reduced lung viral load and pathology, while nasal shedding was reduced but not eliminated, consistent with previous studies^16,17^. VMN delivery of 2-12 C significantly reduced viral load and shedding, while MAD had a transient effect on nasal shedding at day 1 post infection. However, lung viral load and pathology were either absent or significantly reduced in all 2-12C-treated animals.

### Optimisation of contact challenge and duration of co-housing

We next wished to determine whether nasal shedding could be eliminated following a contact influenza challenge that more closely mimics natural infection. In this model, recipient pigs, either treated with 2-12 C or untreated controls, were co-housed with donor pigs previously infected with pH1N1^17^. In this study, contact challenge was conducted in an open barn, where only 3 out of 5 untreated recipients shed the virus and had detectable lung infection. To ensure all recipient control pigs became infected and to generate a robust, reproducible model for assessing mAb treatment, the contact challenge was performed in a high-containment animal facility with regulated temperature and humidity. Furthermore, the donor and recipient pig bedding areas were in enclosures with plastics covers to protect them from the frequent air changes in the room.

**Fig. 3:**
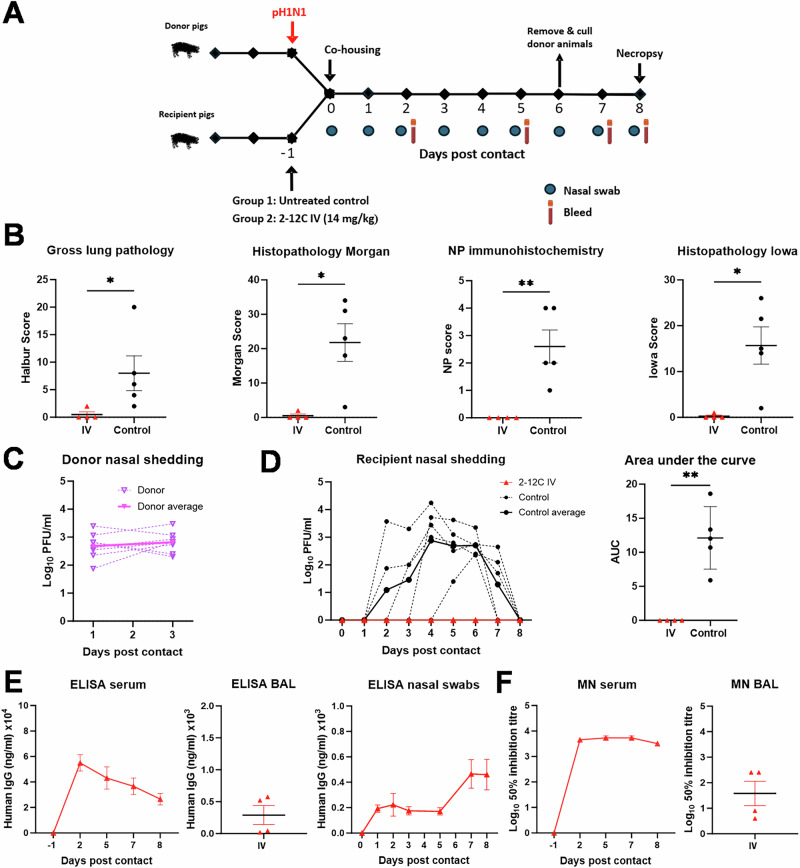
Experiment 3, contact challenge 6 days co-housing. **A** Schematic of experimental design. Recipient pigs were either given 2-12 C mAb intravenously (IV) or were left untreated (control) and then 24 h later were co-housed with previously donor pigs previously infected with pH1N1. The pigs were cohoused for 6 days, and the recipient pigs were culled at day 8 post contact. Nasal swabs and serum were collected at the indicated time points. **B** Lung pathology: gross lung pathology, histopathology Morgan, nucleoprotein (NP) immunohistochemistry and histopathology Iowa scores at day 8 post contact. Virus shedding in nasal swabs from all **C** donor and **D** recipient pigs was analysed by plaque assay at the indicated time points. **E** ELISA titres and **F** neutralising activity in serum, BAL (day 8 post contact) and nasal swabs. Each data point represents an individual pig along (n = 4–5 pigs per group) with the mean ± SEM. Data sets were first assessed for normality and then subjected to an unpaired t-test or a Mann-Whitney U test if they did not pass the normality test. Asterisks denote significance between indicated groups (*p < 0.05, **p < 0.01).

The recipient animals were given either 2-12 C IV at 14 mg/kg or left untreated. One day later the recipient animals were co-housed with donor pigs infected intranasally 1 day previously with pH1N1. The donor pigs were removed after 6 days and culled, and the recipient pigs were culled 2 days later (8 days after contact) to assess virus load and lung pathology (Fig. 3A). Gross and histopathology were abolished or significantly reduced in the 2-12 C treated animals with no NP staining detected by IHC. In contrast, the untreated recipients showed areas of broncho interstitial pneumonia with NP staining present in bronchioli and alveoli (Fig. 3B, Supplementary Fig. S1).

Donor pigs shed virus in the first 3 days confirming the successful infection of all animals and ensuring that recipient pigs were in contact with donors at the peak of viral shedding (Fig. 3C). No virus was detected in the nasal swabs of 2-12 C IV treated recipient pigs (Fig. 3D). In contrast all of five untreated recipient controls shed virus by day 4 post contact. ELISA and MN titres in the 2-12 C-treated group at the time of culling (8 days post contact) were lower compared to the directly challenged pigs which were culled at 4 days post infection (Fig. 3E, F, Table 1 and Figs. 1E, F, 2E, F).

**Fig. 4:**
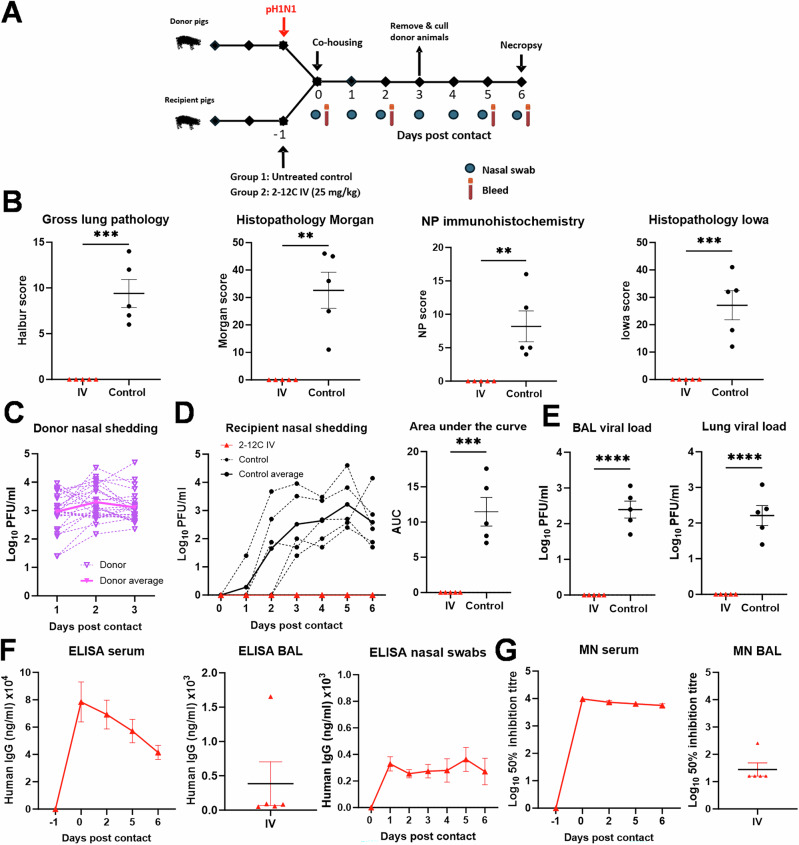
Experiment 4, contact challenge 3 days co-housing. **A** Schematic of experimental design. Recipient pigs were either given 25 mg/kg of 2-12 C mAb intravenously (IV) or were left untreated (control) and then 24 h later were co-housed with previously donor pigs previously infected with pH1N1. The pigs were cohoused for 3 days, and the recipient pigs were culled at day 6 post contact. Nasal swabs and serum were collected at the indicated time points. **B** Lung pathology: gross lung pathology, histopathology Morgan, nucleoprotein (NP) immunohistochemistry and histopathology Iowa scores at day 6 post contact. Virus shedding in nasal swabs from all **C** donor and **D** recipient pigs was analysed by plaque assay at the indicated time points. **E** Viral titres in BAL and accessory lung lobe. **F** ELISA titres in serum, BAL and nasal swabs. **G** Neutralising activity in serum and BAL determined by microneutralisation (MN). Each data point represents an individual pig (n = 5 per group) along with the mean ± SEM. Data sets were first assessed for normality and then subjected to an unpaired t-test. Asterisks denote significance between indicated groups (**p < 0.01, *** p < 0.001, **** p < 0.0001).

It is important to note that the gross lung pathology following direct intranasal challenge with MAD and contact challenge was comparable. Lesions were mostly detected in the cranial and medial lobes with similar severity, although slightly more severe pathology was occasionally observed in directly intranasally infected MAD animals (Supplementary Fig. S2). However, overall, the pattern of pathology was consistent between the two groups. Similarly, the maximum level of viral shedding was comparable between directly infected and contact challenged animals Figs. 1C, 2C, 3C, D, 4C, D.

Having established robust infection in all untreated pigs after six days of co-housing with infected donors, we next investigated whether a shorter, three-day co-housing period would still result in infection of all untreated recipients (Fig. 4A). As before, no gross pathology was detected in the 2-12 C IV treated animals, whereas all untreated control pigs exhibited gross lesions, histopathological changes, and viral presence in the lungs, as confirmed by NP immunohistochemistry (Fig. 4B, Supplementary Fig. S3). Co-housing 2-12C-treated and untreated control pigs with previously infected donors for three days, followed by removal of the donors and an additional three-day observation period, resulted in uniform infection of all control recipient pigs (Fig. 4C, D). By day 2, all untreated control pigs were shedding virus, while none of the 2-12C-treated animals were infected, shed virus, or showed any signs of pathology (Fig. 4B–E). Serum and BAL levels of 2-12 C were 41.5 μg/ml in serum and 0.38 μg/ml in BAL slightly higher than in the 6 days contact challenge above, as a higher dose of 25 mg/kg 2-12 C was administered IV in this experiment (Fig. 4F, G, Table 1).

We have established a robust contact challenge model in which intravenous 2-12 C treatment prevented infection following either 6 or 3 days of co-housing with previously infected donors. The gross lung pathology following direct intranasal challenge with MAD and contact challenge was comparable. The pattern of lung pathology and level of viral shedding was comparable between directly infected and contact challenge animals indicating that results from both models can provide insights into the efficacy of therapies designed to reduce virus transmission.

### Efficacy of mucosal delivery of 2-12 C in the contact challenge model

**Fig. 5:**
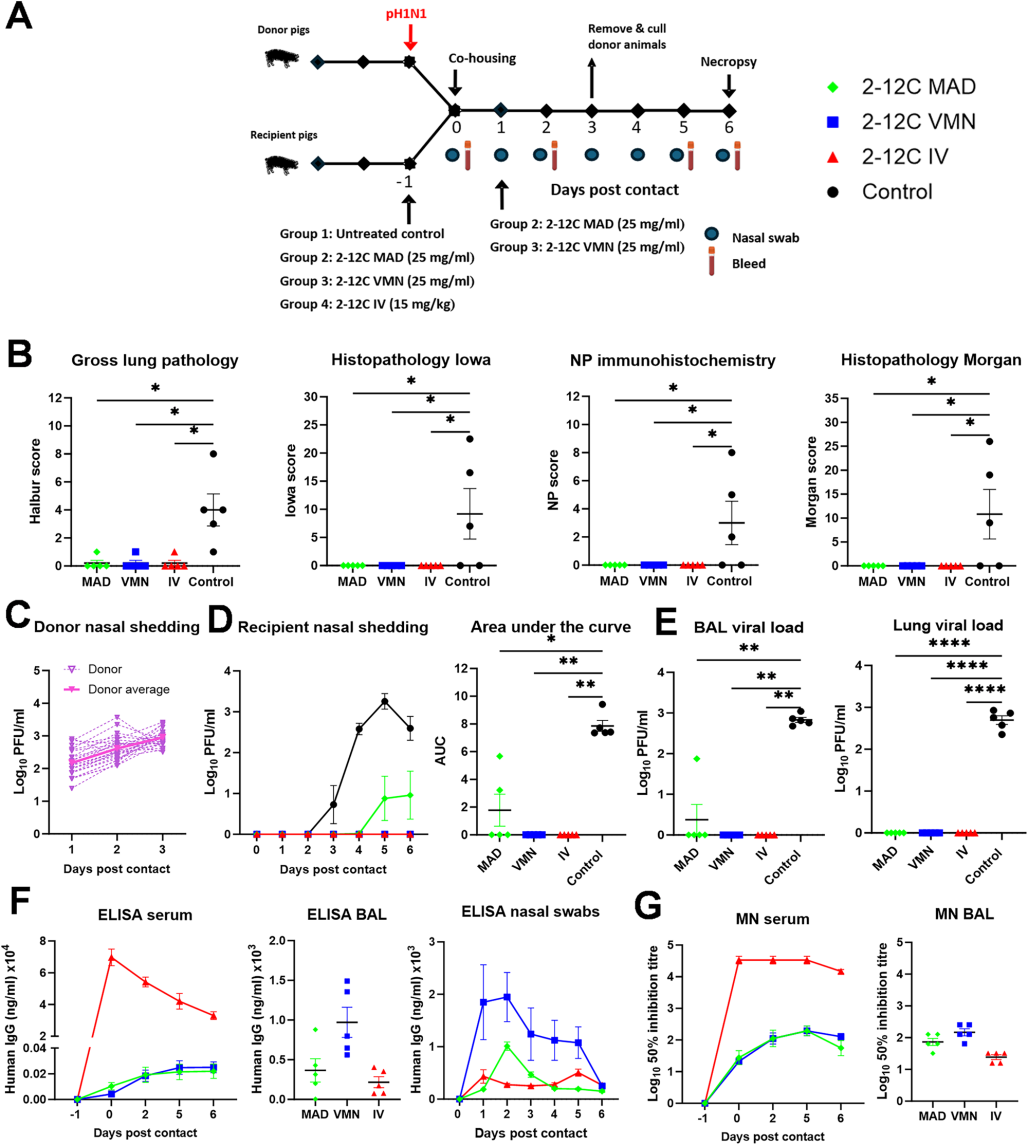
Experiment 5a, contact challenge 3 days co-housing. **A** Schematic of experimental design. Recipient pigs were given 2-12 C mAb either intranasally by mucosal atomization device (MAD) at 25 mg/ml, by aerosol using a vibrating mesh nebuliser (VMN) at 25 mg/ml or intravenously (IV) at 15 mg/kg or were left untreated (control) and then 24 h later were co-housed with donor pigs previously infected with pH1N1. Forty eight hours after the first administration of 2-12 C, the VMN and MAD recipients were administered the same dose of 2-12 C by the same route. The pigs were cohoused for 3 days, and the recipient pigs were culled at day 6 post contact. Nasal swabs and serum were collected at the indicated time points. **B** Lung pathology: gross lung pathology, histopathology Morgan, nucleoprotein (NP) immunohistochemistry and histopathology Iowa scores at day 6 post contact. Virus shedding in nasal swabs from all **C** donor and **D** recipient pigs was analysed by plaque assay at the indicated time points. **E** BAL fluid and lung lysate viral load assessed using plaque assay. **F** ELISA titres and **G** neutralising activity in serum and bronchoalveolar lavage (BAL) fluid (day 6 post contact) and nasal swabs determined by microneutralisation (MN). Each data point represents an individual pig (n = 5 per group) along with the mean ± SEM. Data sets were first assessed for normality and then subjected to either an ordinary one-way ANOVA test with a Dunnett’s multiple comparisons test or a Kruskal-Wallis test with a Dunn’s multiple comparisons test if they did not pass the normality test. Asterisks denote significance between indicated groups (*p < 0.05, ** p < 0.01, **** p < 0.0001).

After establishing a robust contact challenge model, we next assessed the efficacy of mucosal MAD and VMN delivery of 2-12 C. Since the effects of MAD and VMN delivery were more pronounced in the early time points of direct challenge (Fig. 1C), we reasoned that the mucociliary barrier likely cleared 2-12 C from the upper respiratory tract. To overcome this, 2-12 C was administered by MAD or VMN twice, 2 days apart (Fig. 5A). Additional groups received 2-12 C intravenously (single dose) or were left untreated as controls. As before, recipients were co-housed with donor pigs infected with pH1N1 one day previously. The second MAD and VMN doses were given one day after co-housing. Donors and recipients remained together for three days before the donors were removed, and recipients were culled three days later (6 days post contact) (Fig. 5A).

None of the 2-12C-treated pigs showed gross pathology, histopathological changes, or detectable lung virus, as confirmed by NP IHC (Fig. 5B, Supplementary Fig. 4). All donors were productively infected and transmitted the virus to all untreated recipients, who began shedding by day 2 post-contact (Fig. 5C, D). Two of the five MAD-treated 2-12 C recipients started shedding virus from day 4 post-contact, with one of them exhibiting detectable viral load in the BAL (Fig. 5D, E). ELISA and MN serum titres were highest in the 2-12 C IV-treated group, while VMN and MAD titres were nearly two logs lower but comparable. 2-12 C titers were also detected by ELISA in nasal swabs in all treatment groups. Interestingly, MN titres in BAL were comparable between VMN, IV, and MAD groups (Fig. 5F, G).

In this experiment, a group of untreated recipients were included which were co-housed with the infected donors for 2 days and culled 4 days later (Supplementary Fig. S5A). For simplicity and clarity the data for the untreated recipients co-housed for 2 and 3 days is presented separately in Supplementary Fig. S5. No significant differences in pathology were detected between animals housed for 2 or 3 days (Supplementary Fig. S5B). All recipient pigs were infected and shed virus comparable to those co-housed for 3 days, with comparable viral load in BAL and lungs **(**Supplementary Fig. S5C–E).

Overall, these data demonstrate that repeated mucosal delivery by VMN can prevent infection. MAD delivery was less effective, with two of the five pigs shedding virus. IV administration remained the most effective, consistently preventing infection and maintaining strong neutralizing activity in BAL. Two VMN doses of 2-12 C were as efficient as IV in preventing infection in the contact challenge model. Furthermore, a 2-day co-housing period was as effective as 3 days in ensuring that all untreated recipients became infected.

### Threshold shedding of donors for infection of recipients

As the level of viral shedding from donor animals determines the likelihood of infection in recipients, we wished to establish the threshold required for infection in our contact challenge model. Additionally, we sought to determine whether animals treated with 2-12 C and directly challenged with pH1N1, which significantly reduced their viral load, could still transmit the virus to naïve recipients.

**Fig. 6:**
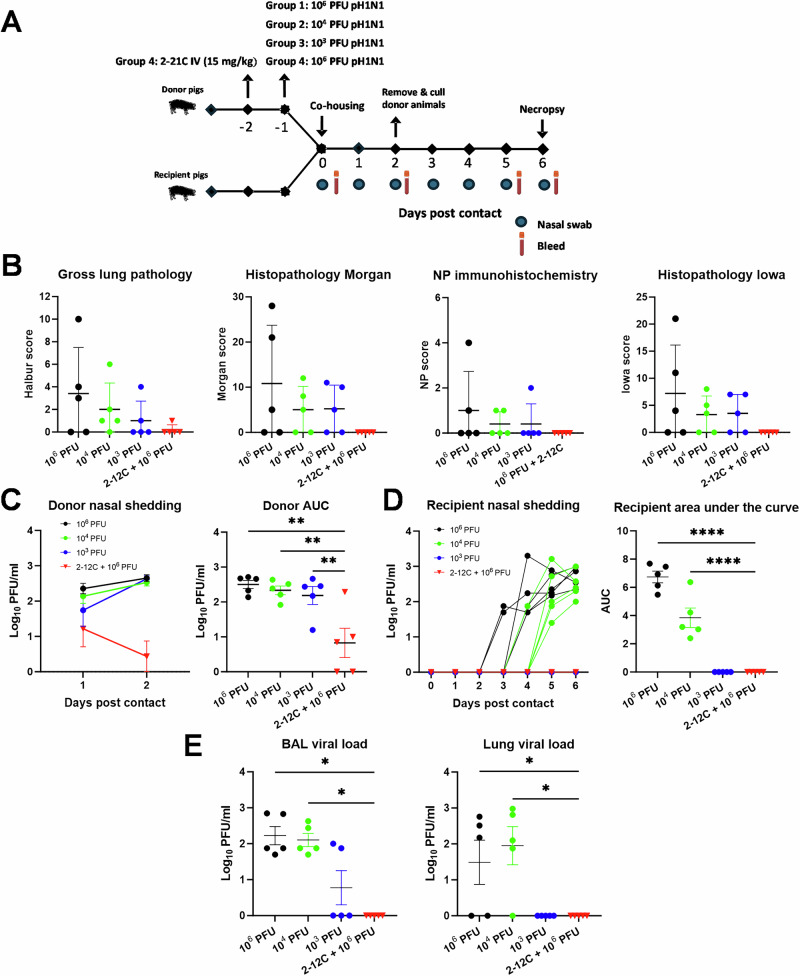
Experiment 6, contact challenge 2 days co-housing. **A** Schematic of experimental design. Groups of donor animals with either 10^6^ PFU, 10^4^ PFU, or 10^3^ PFU of pH1N1. Another group of donors received 2-12 C intravenously at 10 mg/kg and, 24 h later, were inoculated with 10^6^ PFU of pH1N1. Naive recipient animals were co-housed with donors infected one day earlier with 10^6^ PFU, 10^4^ PFU, or 10^3^ PFU, or with 2-12C-treated donors infected with 10^6^ PFU (2-12 C + 10^6^ PFU) for 2 days. Donors were removed and culled after 2 days, and the recipients culled 6 days after contact began. Nasal swabs were collected at the indicated time points. **B** Lung pathology: gross lung pathology, histopathology Morgan, nucleoprotein (NP) immunohistochemistry and histopathology Iowa scores at day 6 post contact. Virus shedding in nasal swabs from all **C** donor and **D** recipient pigs was analysed by plaque assay at the indicated time points. **E** Viral load in BAL (day 6 post contact) and the lung was analysed by plaque assay at day 6 post contact. Each data point represents an individual pig (n = 5 per group) along with the mean ± SEM. Data sets were first assessed for normality and then subjected to an unpaired t-test or a Mann-Whitney U test if they did not pass the normality test. Asterisks denote significance between indicated groups (*p < 0.05, **p < 0.01, **** p < 0.0001).

To investigate this, we infected groups of donor animals with either 10^6^ pfu, 10^4^ pfu, or 10^3^ pfu of pH1N1. Another group of donors received 2-12 C intravenously at 10 mg/kg and, 24 h later, were inoculated with 10^6^ pfu of pH1N1. Naive recipient animals were co-housed with donors infected one day earlier with 10^6^ pfu, 10^4^ pfu, or 10^3^ pfu, or with 2-12C-treated donors infected with 10^6^ pfu (2-12 C + 10^6^ pfu) (Fig. 6A). The donors and recipients were co-housed for two days, after which the donors were removed and culled. The recipients were kept for four additional days and culled six days post-contact.

Lung pathology was detected in the 10^6^ pfu, 10^4^ pfu and 10^3^ pfu donor groups but was absent in 2-12 C + 10^6^ pfu groups (Fig. 6B, Supplementary Fig. 6B). All donors shed comparable levels of virus by day 3 post infection (2 days post contact) despite being infected with different doses (Fig. 6C). The shedding of the 2-12 C treated and 10^6^ pfu infected donors was significantly reduced as consistently shown before with the direct challenge (Figs. 1C and 2C). The recipients co-housed with the 10^6^ pfu and 10^4^ pfu donors shed virus (Fig. 6D), although shedding in the 10^6^ pfu group was detected on day 2 post-contact, whereas in the 10^4^ pfu group, shedding was delayed until day 4 post contact. No shedding was detected in the recipients in the 10^3^ group. However, since the 10^3^ pfu infected donors shed a significant amount of virus, it is likely that the recipients would have eventually become infected if co-housed for a longer period. Two of the animals in the 10^3^ pfu group had detectable virus in the BAL, but none in the lung (Fig. 6E). In contrast, recipients co-housed with donors treated with 2-12 C and infected with 10^6^ pfu did not shed virus and did not have any virus in the lung or BAL.

Because 10⁴ PFU dose of virus in donor pigs was sufficient to infect recipients, we further investigated how the administered volume of the inoculum affected viral shedding and lung pathology. Pigs were inoculated intranasally by MAD with a 10^4^ PFU dose of pH1N1 in three different volumes: 1 ml, 0.5 ml, or 0.25 ml (Supplementary Fig. S7). Pigs infected with 0.5 ml and 0.25 ml volumes showed the highest levels of nasal shedding, while shedding in the 1 ml group was detectable from day 2. All pigs developed lung pathology, with the most pronounced lesions observed in the 1 ml group, consistent with the scintigraphy finding that a 1 ml volume reaches the lungs^14^. The pattern of nasal shedding of 1 ml infected group mimicked best the delayed shedding pattern of the recipients infected by contact.

These results indicate that an infectious dose of 10^4^ pfu in donor animals was sufficient to consistently infect recipients after two days of co-housing. The level and pattern of viral shedding following either direct infection or contact challenge was similar, suggesting that, regardless of the initial viral dose, the virus reaches a consistent replication level. Furthermore, 2-12C-treated, infected animals did not productively transmit the virus, indicating that the treatment reduced viral shedding sufficiently to prevent infection.

## Discussion

In respiratory medicine, the pig model is increasingly recognized as an important bridge between traditional small laboratory animal models and humans^13,18–20^. Pigs exhibit similar clinical manifestations and pathogenesis to humans when infected with influenza viruses making them an excellent model to study immunity to influenza. This similarity extends to the histological structure and physiology of pig lungs which closely resembles that in humans. However, compared to humans, pigs have a more complex nasal and paranasal anatomy and are obligate nasal breathers, whereas humans can breathe through both the nose and mouth. Despite these differences, the pig model remains highly valuable in bridging the gap between small animals and humans for predicting the efficacy of therapeutics in preventing nasal shedding and viral transmission.

We have previously shown that targeting the pig lung is best achieved by aerosolised administration using a VMN. While intranasal MAD delivery resulted in a higher but more variable proportion of the droplets reaching the lung, upper respiratory tract and stomach, VMN delivery by nebuliser provided a more uniform distribution in both lungs and the upper RT^14^. Here we investigated whether delivery of the strongly neutralising 2-12 C mAb to the respiratory tract by VMN or MAD would be more effective compared to intravenous administration in a direct challenge influenza model. High-dose VMN delivery (40 mg/ml in 4 ml volume) was comparable to IV administration (15 mg/kg or 25 mg/kg) in significantly reducing viral shedding, whereas 2-12 C delivered by MAD reduced significantly nasal shedding only on day 1 post infection most likely due to its rapid mucociliary clearance in the RT.

These results indicate that high-dose IV administration of 2-12 C reach the respiratory tract and transudate effectively, directly targeting the site of entry of pathogen without being subjected to mucus clearance. Our findings agree with previous studies showing that intravenous administration of IgG at high enough levels can prevent infection in mice^5^. Interestingly, increasing the IV dose from 15 mg/kg to 25 mg/kg did not enhance efficacy in the direct challenge model. Based on the present and numerous prophylactic studies using intravenous administration of 15 mg/kg 2-12 C in direct challenge pig experiments, a serum concentration of ~130 µg/mL at day 1 post-treatment appears sufficient to significantly reduce viral shedding in the pig direct challenge model^16,17^. In the contact challenge model, a serum concentration of approximately 80 µg/mL at day 1 was sufficient to prevent infection. These values are comparable to the serum concentration for palivizumab in infants against respiratory syncytial virus - from 66 to 153 µg/ml^21^. In contrast it is difficult to establish what concentration of 2-12 C in serum or BAL is required to reduce nasal shedding after VMN delivery, since MAD delivery was inefficient but achieved a higher concentration and greater MN activity in BAL compared to VMN (Fig. 1E, F). However, one should be cautious in interpreting antibody concentrations in BAL, because of the dilution factor and variable recover of lavage fluid.

The contact influenza challenge model is a better approximation of natural exposure, and we demonstrated that co-housing periods of 6 days, 3 days, and 2 days reliably ensured infection in all untreated recipient pigs. Intravenous administration of 2-12 C remained highly effective in preventing infection and by implication transmission in this model. Two doses of 2-12 C administered by VMN, two days apart, were as effective in preventing infection as a single IV dose in the contact challenge model. In contrast, two doses of MAD were less effective, as two out of five pigs shed the virus. While these two pigs were likely capable of transmitting infection, the study was terminated at day 6 post-contact, preventing further assessment. All 2-12 C IV, MAD, and VMN-treated animals showed comparable antibody titres in BAL of ~0.3 µg/ml at day 6 post-challenge, suggesting this concentration may be sufficient to predict efficacy in the contact challenge model.

It is important to note that gross lung pathology following direct intranasal challenge by MAD or contact challenge was comparable with lesion localised in the cranial and medial lobes. This indicates that, regardless of the presumed much lower viral dose in contact challenge, the virus still reaches and replicates in the lungs. Similarly, the peak level of viral shedding was comparable between directly infected and contact-challenged animals. However, in contact challenged pigs, viral load and shedding increased gradually, potentially providing a wider therapeutic window compared to the immediate high-dose exposure in direct challenged animals. This pattern of nasal shedding was also observed in pigs directly infected with 1 ml of 10^4^ pfu pH1N1. Despite its physiological relevance, contact challenge is resource-intensive, requiring twice the number of animals, substantial labour, and specialised animal facilities with controlled temperature and humidity to ensure consistent results. Given that animals directly infected MAD exhibit similar lung pathology and infection dynamics to contact-challenged animals, this model offers a reasonable approximation of natural exposure. Our results strongly suggest that a significant reduction in viral load in the direct challenge model is likely to indicate that infection might be prevented in the contact challenge model.

It is also important to note that as we show that preventing transmission is not easily to achieved in the pig model, that delivery of mAbs that did not prevent nasal shedding but abolished viral load in the lung and BAL and may, may still be a very useful therapeutic strategy to prevent severe disease.

An important question is whether to target only the upper or the entire respiratory tract, including the lungs. In humans, two main inhalation approaches exist: (i) intranasal inhalation, which delivers therapeutics to the upper RT via the nose, and (ii) pulmonary inhalation, which delivers drugs to the lungs via the airways. For optimal nasal deposition, particle aerodynamic diameters should range between 10–100 µm, whereas effective lung deposition requires particles between 1 and 5 µm. While nasal sprays targeting the upper RT are non-invasive, offer rapid onset of action, and ensure high patient compliance, they may not reach all infected cells. For example, both influenza and SARS-CoV-2 infect both the nasal epithelium and can spread to the lung. In our pig model, both VMN and MAD targeted the upper and lower respiratory tract although with different distributions. However, VMN was clearly more protective. It remains to be determined whether restricting therapeutics to the upper RT in pigs will be effective.

Another key question is which route is more cost-effective in terms of the delivered dose. In this study, we tested 15 mg/kg doses of intravenous 2-12 C in pigs with an average weight of 10.4 kg (experiment 5), equating to 156 mg per pig. Two doses of 2-12 C were used for MAD and VMN delivery to maintain protection in the contact challenge experiment, requiring 150 mg of mAb per animal. However, given that VMN delivers only ~30% of the dose to the respiratory tract it appears that it is very effective in terms of the mAb actually deposited in the animal. It will be important to determine the pharmacokinetic and duration of protection following respiratory delivery of mAbs. The pharmacokinetics of intravenous 2-12 C suggest that protection mediated by 15 mg/kg would likely be maintained for an extended period^22^.

Future advances in long-term delivery platforms and modifications to circumvent antibody clearance will further enhance mAbs half-life. Already alternative strategies for in vivo antibody delivery, including gene transfer via DNA, RNA, viral vectors, polymer conjugation, and Fc modifications, have shown promise in maintaining stable and prolonged antibody expression in host tissues. We propose that the pig direct and contact challenge models are powerful tools to evaluate transmission blocking by novel therapeutics.

## Materials and methods

### 2-12 C mAb preparation

The anti-influenza HA1 human IgG1 mAb 2-12 C was produced by Absolute Antibody Ltd (Redcar, UK). It was dissolved in 25 mM histidine, 150 mM NaCl, and 0.02% Tween 20 (pH 6) diluent.

### Influenza infection of pigs

All experiments were approved by the ethical review processes at the Pirbright Institute and Animal and Plant Health Agency (APHA) and were conducted in accordance with the UK government Animals (Scientific procedures) Act 1986, supported by project licence PP2064443. Seven influenza challenge experiments were carried out, three direct challenges and four contact challenges (Table 1). For all studies, 4–5-week-old female Large white x Landrace pigs were obtained from a commercial high-health status herd. The pigs were screened by ELISA for the absence of serum antibodies against A/swine/England/1353/2009 (pH1N1). In all experiments, animals acclimatized for at least 7 days and were randomized into different groups and pens using Excel by the animal services staff. Researchers processing the samples were only aware of the pig numbers, not the group assignments. The pathologists were blinded to the group allocation when assessing the samples for gross pathology during post-mortem examination and subsequently during the histopathological assessment. All conditions were kept the same between groups. Viral shedding was the main outcome variable for the studies. From previous experiments, the standard deviation in shedding is 0.8 log₁₀ pfu/ml. A reduction in mean viral shedding of 2 log_10_ pfu/ml is sufficient to show that treatment with the mAb has equivalent reduction to current influenza vaccines^23,24^. Using this difference and standard deviation a group size of five or six pigs is required to detect a difference between groups with >79% or 90% power respectively at 95% confidence (one-way ANOVA, six groups; Minitab 20). This design allows assessment of the effects of dose and delivery route with primary measures viral load as well as lung pathology.

### First experiment (direct challenge I)

Animal experiment was conducted in APHA - ethical approval PP2064443-1-004, 6th August 2024. Twenty four pigs (average weight 10.8 kg) were randomized into four groups of six pigs as follows: 1) 2-12 C administered intravenously (IV) at 15 mg/kg into the ear vein, 2) 2-12 C (3 ml of 15 mg/ml, 1.5 ml per nostril) administered intranasally by mucosal atomization device (MAD, Medtree, UK), 3) 2-12 C (4 ml of 15 mg/ml) administered by aerosol using vibrating mesh nebuliser (VMN, Aerogen), and 4) untreated controls. For the 2-12 C mAb intravenous, MAD and VMN administration pigs were sedated with 1.5 mg/kg Zoletil (Virbac, UK) and 0.04 mg/kg medetomidine (Domitor, Orion Pharma, Finland). Twenty-four hours after 2-12 C administration, all animals were intranasally inoculated by MAD with 3 ×10^6^ pfu of pH1N1 MDCK grown in 2 ml (1 ml per nostril) without sedation. Daily nasal swabs were collected for 4 days, and blood samples were collected at -1, 1-, 3-, and 4-days post infection (DPI). The pigs were humanely killed 4 days post pH1N1 infection with an overdose of pentobarbital sodium anaesthetic, confirmed by the permanent cessation of circulation. Lung pathology was assessed, and blood, bronchoalveolar lavage (BAL), and accessory lung were collected for assessment of viral load and antibody titres.

### Second experiment (direct challenge II)

Animal experiment was conducted in APHA - ethical approval PP2064443-1-003v1 23^rd^ Feb 2024. Fifteen pigs (average weight 11.6 kg) were randomized into three groups of five pigs as follows: 1) 2-12 C administered IV at 40 mg/kg following sedation as above, 2) 2-12 C at 25 mg/ml (4 ml) administered by VMN as above, and 3) untreated controls. Twenty-four hours after 2-12 C administration, all animals were intranasally inoculated by MAD with 3 ×10^6^ PFU of pH1N1 in 2 ml (1 ml per nostril). Daily nasal swabs were collected for 4 days, and blood samples were collected at -1, 0, 1, 3, and 4 DPI. At 4 days post pH1N1 infection, the pigs were humanely euthanized with an overdose of pentobarbital sodium anaesthetic, confirmed by the permanent cessation of circulation. The lung pathology was assessed, and blood, BAL fluid, and accessory lung were collected.

### Third experiment (contact challenge 6 days co-housing)

Contact challenges were performed in the high containment isolation unit at the Pirbright Institute at 19 °C and relative humidity of 55%—approved by animal facility team; named veterinary surgeon (NVS), named animal care and welfare officer (NACWO), animal facility manger, in house statistician, home office liaison contact (HOLC) and named information officer (HIO) officer, AR001352, 18th August 2023. Twenty pigs (average weight 12.2 kg) were split into two groups of ten donor pigs and ten recipient pigs. The recipient pigs were further randomized into two groups of 5 pigs as follows: 1) 2-12 C 14 mg/ml administered IV into the ear vein following sedation with a 1.5 mg/kg Zoletil and 3 mg/kg Stresnil (Elanco UK AH Limited), 2) untreated controls. The donor pigs were infected intranasally by MAD with 5 ×10^6^ PFU pH1N1 (1 ml per nostril). After 24 h the five recipient 2-12 C pigs were put in contact with five donor pigs infected 24 h previously with pH1N1. Similarly, the five control untreated recipient pigs were put in contact in a separate room with five previously infected donor pigs. After 6 days of cohousing the donors were removed and culled. The recipient pigs were humanely culled 2 days later (8 days after cohousing) with an overdose of pentobarbital sodium anaesthetic, confirmed by the permanent cessation of circulation. Lung pathology was assessed, and samples of blood, BAL, and accessory lung tissue were collected for evaluation of viral load and antibody titers. Nasal swabs were taken daily from all pigs, and serum was collected at 2-, 5-, 7-, and 8- days post contact (DPC) from the recipient group only.

### Fourth experiment (contact challenge 3 days co-housing)

Contact challenges were performed in the high containment isolation unit at the Pirbright Institute—approved by animal facility team (as above), AR001405, 29^th^ April 2024. Twenty pigs (average weight 11.7 kg) were randomised and split into two groups of ten donor and ten recipient pigs. The recipient pigs were further randomized into two groups of five 1) 2-12 C administered IV at 25 mg/kg into the ear vein following sedation as in experiment 3 and 2) untreated controls. The donor group was infected with 5 ×10^6^ PFU/ml pH1N1 virus. Twenty-four hours after 2-12 C administration or pH1N1 challenge, the recipients and donors were cohoused together. After 3 days of co-chousing the donor pigs were removed. The recipient pigs were humanely culled 6 days post contact with an overdose of pentobarbital sodium anaesthetic, confirmed by the permanent cessation of circulation. The lung pathology was assessed, and blood, BAL and accessory lung were collected. Nasal swabs were taken daily from all pigs, and serum was collected at 0-, 2-, 5-, and 6- DPC from the recipient group only.

### Fifth experiment (contact challenge 3- and 2-days co-housing)

Contact challenges were performed in the high containment isolation unit at the Pirbright Institute—approved by animal facility team (as above), AR001423, 27th Sep 2024. Fifty pigs (average weight 10.4 kg) were split into two groups of twenty-five donor and twenty-five recipient pigs. The recipient pigs were randomized into four groups of five pigs as follows: 1) 2-12 C administered IV at 15 mg/kg into the ear vein, 2) 2-12 C administered by MAD (25 mg/ml, 1.5 ml per nostril) following sedation as in experiment 3, 3) 2-12 C administered by VMN (25 mg/ml, 3 ml), and 4) untreated controls. VMN and MAD administration as repeated after 48 h by the same route and dose. The 2-12 C administration was performed following sedation as in experiment 3. The donor group was infected with 5 ×10^6^ PFU pH1N1. Twenty-four hours after 2-12 C treatment or pH1N1 challenge, each group of recipients was put in contact with five pH1N1 infected donors. Recipients and donors were cohoused together for 3 days, at which point the donor pigs were removed. The recipient pigs were kept for 3 more days and humanely killed 6 days post contact (labelled as Experiment 5a - 3 days co-housing). One untreated recipient group was cohoused with 5 donor pigs for 2 days only to compare the efficacy of contact infection between 2- and 3-days co-housing (labelled as Experiment 5b - 2 days co-housing). The recipient pigs were humanely culled 6 days post contact with an overdose of pentobarbital sodium anesthetic, confirmed by the permanent cessation of circulation. Lung pathology was assessed, and samples of blood, BAL, and accessory lung tissue were collected for evaluation of viral load and antibody titers. Nasal swabs were taken daily from all pigs, and serum was collected at 0-, 2-, 5-, and 6-DPC from the recipient group only.

### Sixth experiment (contact challenge 2- days co-housing)

Contact challenges were performed in the high containment isolation unit at the Pirbright Institute - approved by animal facility team (as above), AR001465, 8th January 2025. Forty pigs (average weight 16 kg) were split into two groups of 20 donors and 20 recipients. The donor group were randomised into 4 groups of five animals. One group was given 2-12 C intravenously at 10 mg/kg. Twenty-four hours later all four groups of donors were intranasally inoculated using MAD with pH1N1 with the following doses: 1) 1 ×10^6^ pfu; 2) 1 ×10^4^ pfu; 3) 1 ×10^3^ pfu. 4) The group treated with 2-12 C was inoculated with 1 ×10^6^ pfu pH1N1. Twenty-four hours after the pH1N1 challenge, the recipients and donors were cohoused together. After 2 days of co-chousing the donor pigs were removed. The recipient pigs were humanely culled 6 days post contact with an overdose of pentobarbital sodium anaesthetic, confirmed by the permanent cessation of circulation. Lung pathology was assessed, and samples of blood, BAL, and accessory lung tissue were collected for evaluation of viral load and antibody titers. Nasal swabs were taken daily from all pigs, and serum was collected at 0-, 2- DPC from the 2-12 C-treated group only.

### Seventh experiment (direct challenge, different volumes of viral inoculum)

The direct challenges were performed in the high containment isolation unit at the Pirbright Institute - approved by animal facility team (as above), AR001496, 18th March 2025. This was an exploratory pilot study with 3 or 4 pigs per group. Ten pigs (average weight 10.6 kg) were randomly allocated into three groups and infected with 10^4^ pfu pH1N1 delivered either in 1 ml (3 pigs), 0.5 ml (3 pigs) and 0.25 ml (4 pigs). Daily nasal swabs were collected for 4 days. At 4 days post pH1N1 infection, the pigs were humanely euthanized with an overdose of pentobarbital sodium anaesthetic, confirmed by the permanent cessation of circulation. Lung pathology was assessed, and samples of blood, BAL, and accessory lung tissue were collected for evaluation of viral load and antibody titers.

Clinical signs, including temperature, coughing, breathing status, appetite, nasal discharge, and behavioural change, were monitored daily after the pH1N1 challenge in all experiments. The observed signs were mild, and none of the pigs developed moderate or severe disease.

### Pathological and histopathological examination of lungs

Gross pathology and histopathological analysis were performed at postmortem as described previously^25^. In brief, the lungs were removed, and the dorsal and ventral aspects of the lungs were photographed. Macroscopic pathology was blindly scored by a veterinary pathologist as previously reported^26^. Lung tissue samples were taken from the right cranial, middle, and caudal lung lobes from the right side of the lung and immersed into 10% neutral-buffered formalin for histological processing. The formalin-fixed tissue was embedded in paraffin wax, cut into 4-mm sections and routinely stained with H&E and immunohistochemistry against influenza A virus nucleoprotein (NP). Lung histopathology and IHC staining were assessed by a veterinary pathologist blinded to the treatment group. Pulmonary histopathology was scored (“Morgan”) using five parameters (airway inflammation, alveolar exudates, septal inflammation, necrosis of the bronchiolar epithelium and perivascular/bronchiolar cuffing) using a five-point scale of 0–4, the sum of which gave a total score ranging from 0–20 per lobe^27^. A mean score for the three lung lobes was calculated for each animal. Another scoring system “Iowa” was used to score the individual lung lobes and also considering the amount of NP present in the samples^28^.

### Virus titration

Viral titres in nasal swabs, accessory lung lobe and BAL fluid were determined by plaque assay using Madi-Darby canine kidney (MDCK) cells. Samples were serially diluted in Dulbecco’s Modified Eagle Medium (DMEM), before being overlayed on confluent MDCK cells. After incubation at 37 °C at 5% CO_2_ for 1 hour, plates were washed with PBS and overlayed with medium consisting of 1× Minimum Essential Medium Eagle (Sigma-Aldrich). 0.3% BSA, 2.86 mM L-Glutamine, 0.3% sodium bicarbonate, 14 mM 4-(2-hydroxyethyl)-1-piperazineethanesulfonic acid (HEPES), 0.007% dextran, 100 IU/ml penicillin, 100 μg/ml streptomycin and 0.6% agar (Oxoid). After a further incubation at 37 °C at 5% CO_2_ for 72 h, the overlay was removed and 0.1% crystal violet (Sigma-Aldrich) in 20% methanol (Sigma-Aldrich) was used to stain the cells and count the plaques. Plaque forming units per mL (PFU/ml) were calculated as the average number of plaques divided by the dilution factor.

### Tissue sample processing

Serum was collected using BD Vacutainer SST™ Serum Separation Tubes which were centrifuged for 15 mins at 1000 × *g*. Two nasal swabs, one from of each nostril, were taken at the time points indicated and placed in 2 ml of virus transport medium (VTM) consisting of medium 199 (Sigma-Aldrich) supplemented with 0.5% BSA, 100 IU/ml penicillin, 100 μg/ml streptomycin, 0.035% sodium bicarbonate, 25 mM HEPES and 0.25% mg/ml nystatin. The samples were vortexed, centrifuged to remove debris, and stored at −80 °C for subsequent virus titration.

BAL fluid was collected using 0.3% BSA in PBS which was added into the left lung and then collected in falcon tubes. BAL fluid was then centrifuged at 500 × *g* for 5 mins, with supernatant removed and frozen until use. Accessory lung lobes were collected from all pigs at postmortem and frozen at −80 °C. After thawing, 2 g of tissue, extracted uniformly from across the entire lobe, was added to 9 ml of RPMI media and homogenized using a Miltenyi Cell Dissociator. An additional 9 ml of RPMI media was then added before the cell suspension was centrifuged at 500 × *g* for 5 min. The supernatant was removed, aliquoted and frozen for analysis of viral load by plaque assay and antibody titres.

### ELISA and microneutralisation

Serum, BAL fluid and nasal swab antibody titres were assessed by ELISA against recombinant HA protein of A/Eng/195/2009 hemagglutinin as before^29^. Briefly, serum, BAL fluid and nasal swabs were heat inactivated at 56 °C for 30 mins and diluted in buffer (0.05% Tween 20 in PBS). HA coated plates were blocked using 4% (w/v) milk powder (Marvel) in PBS containing 0.05% Tween 20 (Sigma-Aldrich) before the sera samples were incubated with HA-coated plates in duplicate for 1 hour at RT for serum and BAL fluid and overnight for nasal swabs. The plates were washed twice and incubated with HRP conjugated goat anti-human IgG-Fc antibody HRP (Bethyl laboratories, A80-304P) for 1 hour at RT and developed using 3,3′,5,5′-tetra-methylbenzidine (TMB) substrate. The plates were read at 450 nm and 630 nm (reference wavelength) with the Absorbance Microplate Reader (BioTek, Swindon, UK). The serum concentration was interpolated from the standard curve using a sigmoidal four-parameter logistic curve fit for the log of the concentration.

Neutralising antibody titres were determined in serum and BAL fluid using microneutralisation assay as before^16^. In brief, serum and BAL fluid that were heat inactivated at 56°C for 30 mins and were serially diluted before being incubated with an equal volume of pH1N1. After 2 h, MDCK SIAT-1 cells at 3 ×10^4^ cells per well were added and then incubated for a further 18 h. The cell monolayer was fixed with 4% paraformaldehyde and permeabilised with 0.05% Triton-X100 and 20 mM glycine and then stained with mouse anti-NP IAV IgG1 (Clone AA5H, Bio-Rad Laboratories) followed by goat anti-mouse HRP secondary antibody (DAKO). After addition of the TMB substrate, the reaction was stopped with 1 M sulfuric acid, and absorbance was measured at 450 and 570 nm (reference wavelength) on the Absorbance Microplate Reader (BioTek). The 50% inhibition titre was defined as the final dilution of serum that caused ≥50% reduction in NP expression.

### Statistical analysis

Statistical analyses were performed using GraphPad Prism 10.0.2 (GraphPad software, San Diego, CA, USA). Data sets were first assessed for normality and then subjected to either a t-test or one-way or two-way ANOVA test and a post-hoc Tukey’s multiple comparisons test or Dunnett’s multiple comparisons test, or a Kruskal-Wallis test and a post-hoc Dunn’s multiple comparisons test if they did not pass the normality test. Significant differences were presented on each graph (*p < 0.05, **p < 0.01, ***p < 0.001, ****p < 0.0001). No data points were excluded from the analysis.

## Supplementary information

Supplementary Figures 5th sep

## Data Availability

The datasets generated and/or analysed during the current study are provided as a source data file. Any further data may be provided by contacting the corresponding author upon reasonable request.
