# Supplementary material for: Aerosol and intranasal delivery of monoclonal antibodies to prevent transmission in pig influenza infection models
Source: NPJ Vaccines. 2025 Nov 5;10:226. doi: 10.1038/s41541-025-01277-9 (PMC12589635; doi:10.1038/s41541-025-01277-9)
Supplement: Supplementary file 1 — Supplementary Figures 5th sep [file 41541_2025_1277_MOESM1_ESM.pdf]

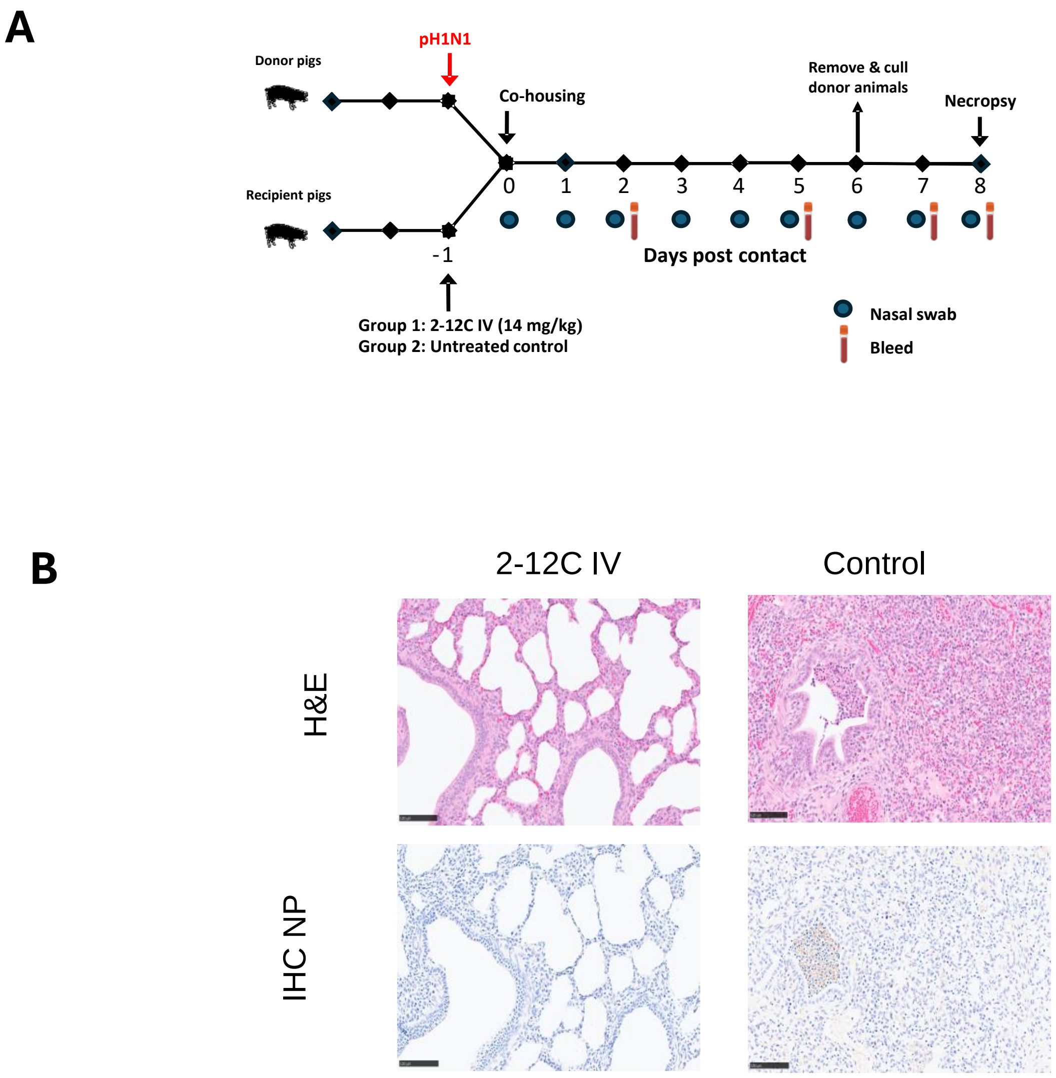

Supplementary Figure 1 . Experiment 3, contact challenge 6 days co-housing. (A) Schematic of experimental design. (B) Representative histopathology (H&E staining), and immunohistochemical NP staining for each group.

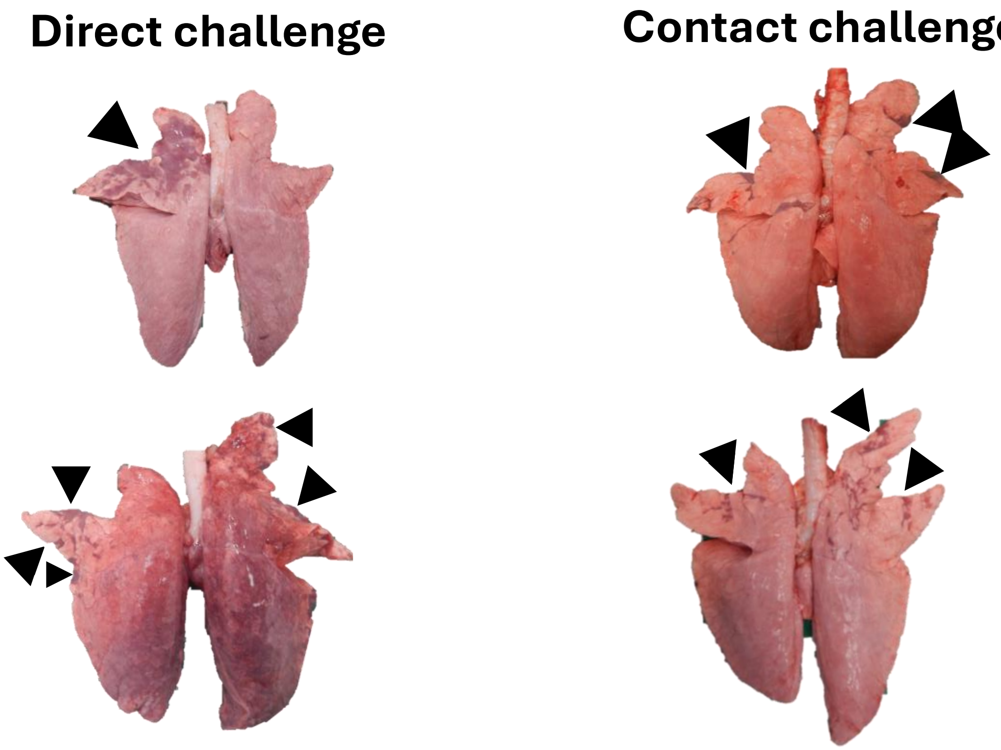

Supplementary Figure 2: Gross lung pathology after direct or contact challenge with Influenza A virus. Dorsal view of four representative pigs (two from each group) showing multifocal areas of broncho-interstitial pneumonia affecting mainly the cranial and middle lobes (arrows). A similar distribution and severity is observed in both groups.

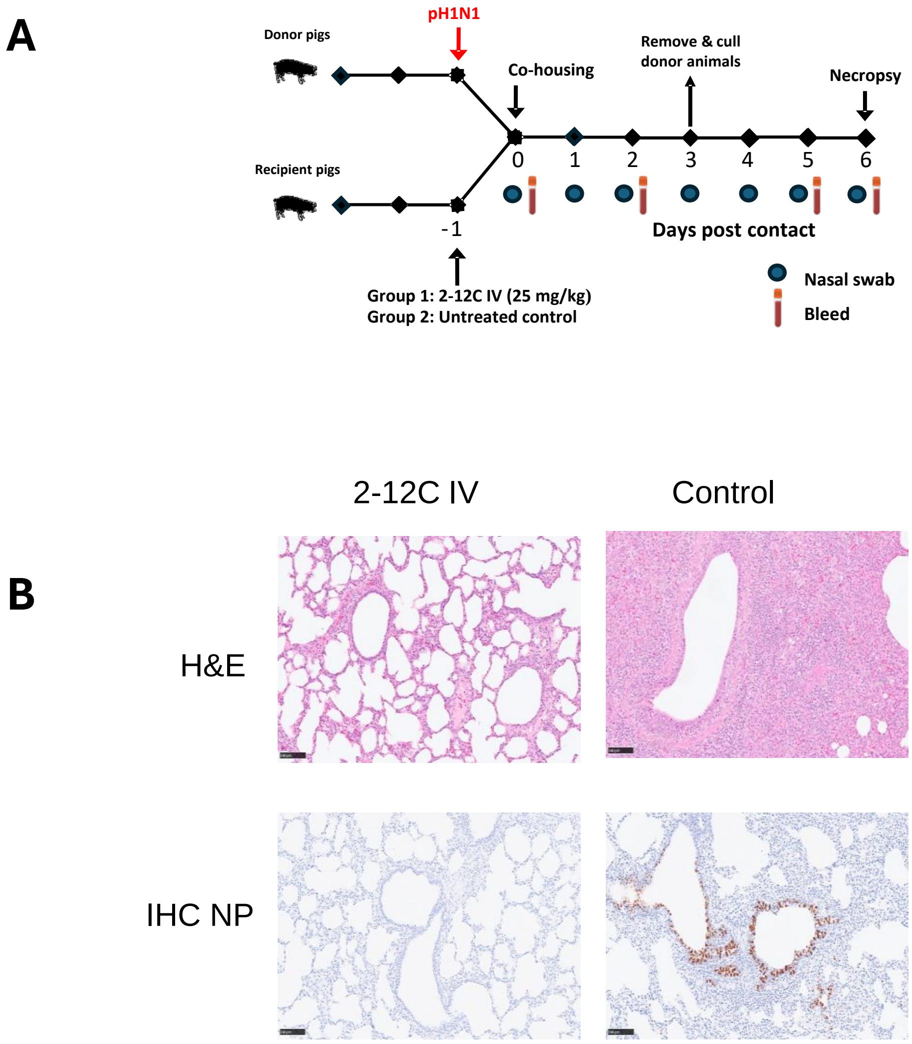

Supplementary Figure 3. Experiment 4, contact challenge 3 days co-housing (A) Schematic of experimental design. (B) Representative histopathology (H&E staining), and immunohistochemical NP staining for each group.

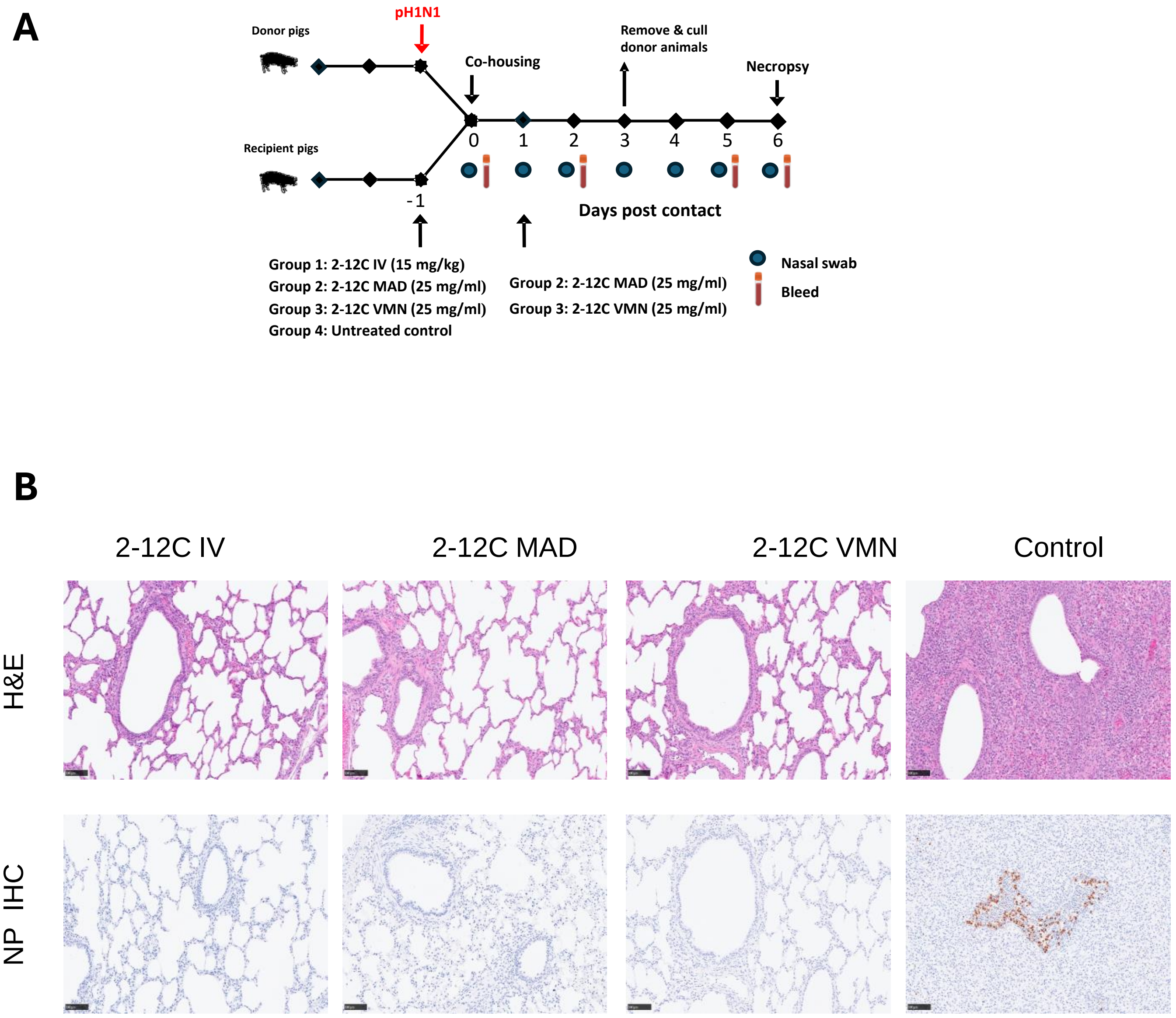

Supplementary Figure 4. Experiment 5a, contact challenge 3 days co-housing (A) Schematic of experimental design. (B) Representative histopathology (H&E staining), and immunohistochemical NP staining for each group.

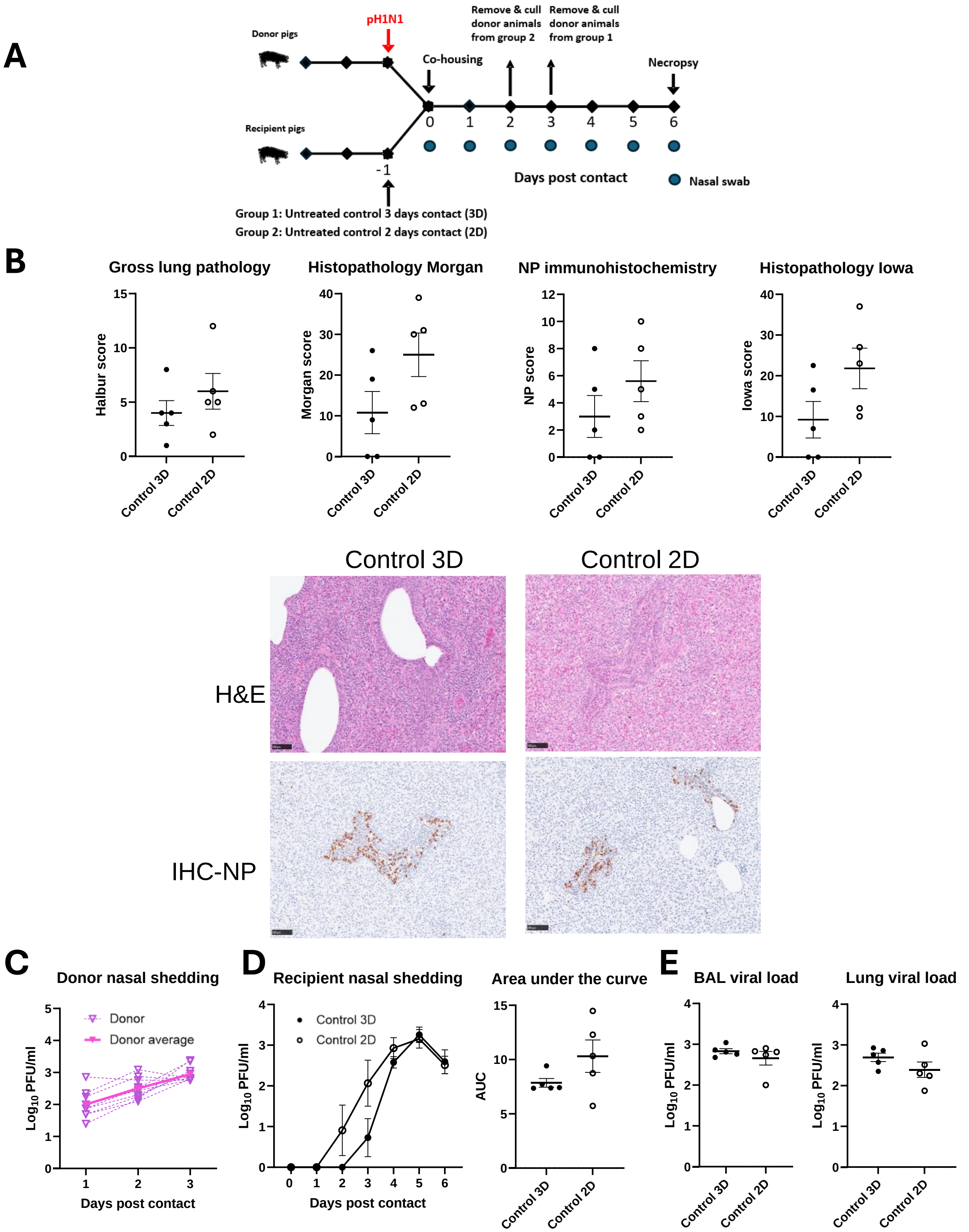

Supplementary Figure 5: Experiment 5b, contact challenge 2 days. (A) Schematic of experimental design. Untreated recipient pigs were co-housed with pigs previously infected with pH1N1 for either 2 (D2) or 3 days (D3). The recipient pigs were culled after further 3 or 4 days, respectively, and the donors were culled 6 days after contact began. Nasal swabs were collected at the indicated time points. **(B)** Lung pathology: gross lung pathology, histopathology Morgan, nucleoprotein (NP) immunohistochemistry and histopathology Iowa scores at day 6 post contact. Representative histopathology (H&E staining), and immunohistochemical NP staining for each group. Virus shedding in nasal swabs from all **(C)** donor and **(D)** recipient pigs was analysed by plaque assay at the indicated time points. BAL and lung viral load were determined at the time of the culls (6 days post contact) **(E)**. Each data point represents an individual pig (n=5 per group) along with the mean±SEM. Data sets were first assessed for normality and then subjected to an unpaired t-test or a Mann-Whitney U test if they did not pass the normality test.

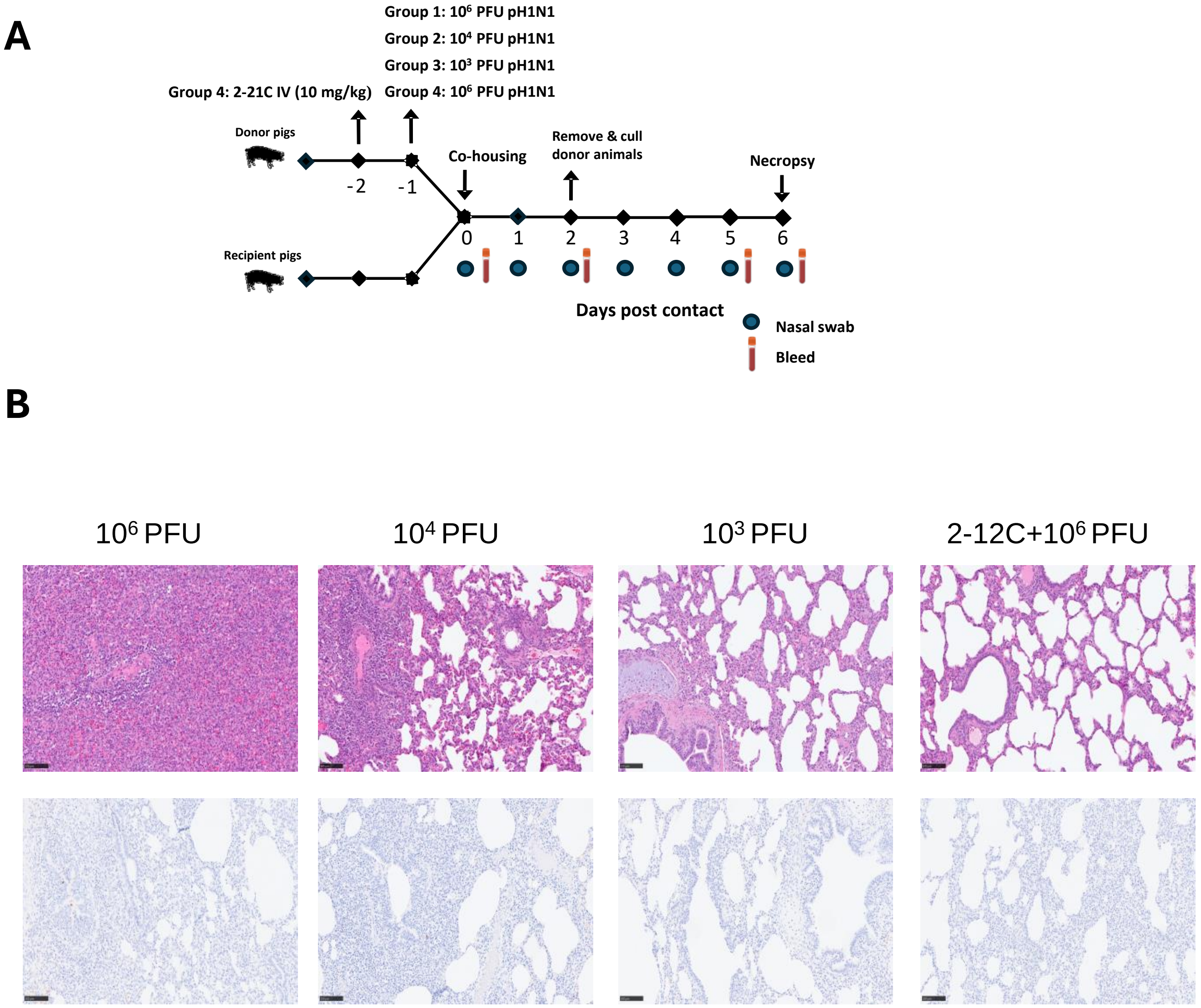

Supplementary Figure 6. Experiment 6, contact challenge 2 days co-housing (A) Schematic of experimental design. (B) Representative histopathology (H&E staining), and immunohistochemical NP staining for each group.

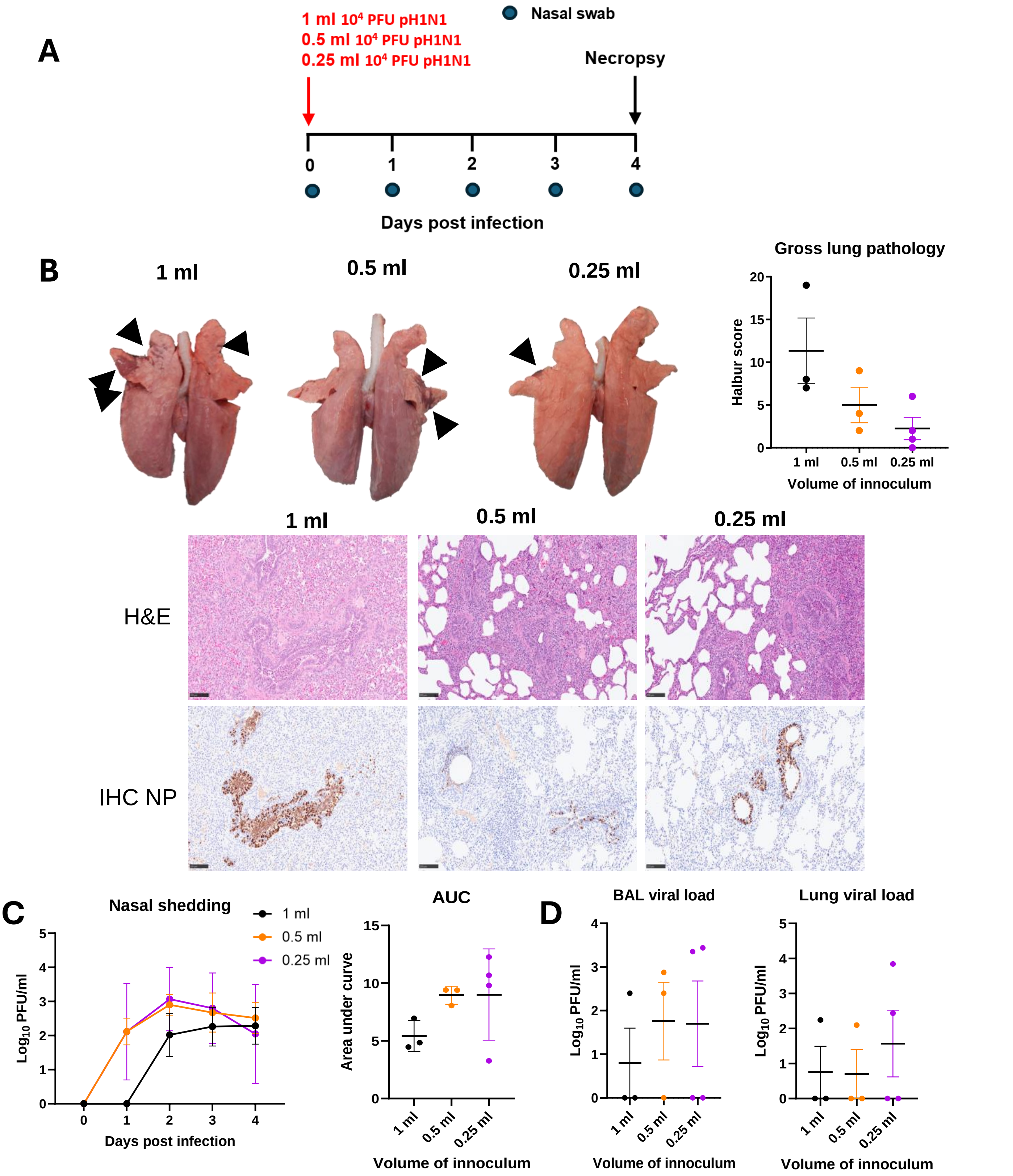

Supplementary Figure 7: Experiment 7, direct challenge with different volumes of virus inoculum. (A) Schematic of experimental design. Pigs were intranasally inoculated with 10^4 pfu pH1N1 administered in 1ml, 0.5ml or 0.25ml using MAD. Nasal swabs were collected at the indicated time points. Necropsy was performed at 4 days post infection. **(B)** Lung pathology: **i**mages and gross lung pathology, histopathology Morgan, nucleoprotein (NP) immunohistochemistry and histopathology Iowa scores at day 4 post infection, Representative histopathology (H&E staining), and immunohistochemical NP staining for each group **(C)** Virus shedding in nasal swabs from all pigs were analysed by plaque assay at the indicated time points. **(D)** BAL and lung viral load were determined at the time of the cull (day 4). Each data point represents an individual pig (n=3-4 per group) along with the mean±SEM. Data sets were first assessed for normality and then subjected to an unpaired t-test or a Mann-Whitney U test if they did not pass the normality test.
